# Nonfamilial VACTERL-H Syndrome in a Dizygotic Twin: Prenatal Ultrasound and Postnatal 3D CT Findings

**DOI:** 10.3390/medicina59081387

**Published:** 2023-07-28

**Authors:** Seol Young Hong, Soo Jung Kim, Mi-Hye Park, Kyung A. Lee

**Affiliations:** Department of Obstetrics and Gynecology, Ewha Womans University College of Medicine, Ewha Womans University Seoul Hospital, Seoul 07804, Republic of Korea; eley1002@gmail.com (S.Y.H.); bossksj25@gmail.com (S.J.K.); ewhapmh@ewha.ac.kr (M.-H.P.)

**Keywords:** VACTERL-H, prenatal diagnosis, dizygotic twin

## Abstract

*Background*: VACTERL association is a widely known congenital malformation that includes vertebral, anal, cardiac, tracheoesophageal, renal, and limb anomalies. Patients with VACTERL and hydrocephalus appear to form a distinct group, both genetically and phenotypically, and their condition has been called VACTERL-H syndrome. Most cases of VACTERL-H have been reported postnatally, as VACTER-H syndrome is difficult to diagnose prenatally. *Case Presentation:* Here, we report a case of VACTERL-H syndrome in a dichorionic and diamniotic twin diagnosed prenatally by ultrasonography and confirmed postnatally by three-dimensional computed tomography (3D CT). A 34-year-old multiparous female was referred to our institution at 31 + 3 weeks gestation for suspected fetal ventriculomegaly. Detailed examinations using two-dimensional and Doppler ultrasounds revealed hydrocephalus, bilateral dysplastic upper arms, radial aplasia, unilateral pulmonary agenesis, dextrocardia with right atrial enlargement, a unilateral hypoplastic ectopic kidney, a single umbilical artery, a tracheoesophageal fistula with a small stomach, polyhydramnios, and anal atresia. Findings from the postnatal 3D CT aligned with the prenatal diagnosis, showing upper-limb agenesis, dextrocardia with pulmonary hypoplasia, tracheoesophageal fistula, imperforate anus, and colon dilatation. The affected 1390-g male twin had an unaffected 1890-g female twin sister and a healthy 6-year-old brother. *Conclusions*: Upon encountering fetuses with multiple anomalies, including ventriculomegaly, a small stomach with polyhydramnios, an abnormally positioned heart, and upper-limb abnormalities, clinicians should perform systematic ultrasonographic examinations to detect associated anomalies and be aware of VACTERL-H syndrome.

## 1. Introduction

A VATER, VACTER, or VACTERL association occurs in one of 10,000 to 40,000 infants and includes at least three of the following core abnormalities, which occur AT these frequencies: vertebral defects (V; fusion, hemivertebrae, and scoliosis), 60% to 80%, anorectal atresia (A), 60% to 90%, cardiac defects (C; ventricular septal defect, tetralogy of Fallot, transposition of the great arteries, and other cardiovascular anomalies), 40% to 80%, tracheoesophageal fistula (TE), 50% to 80%, renal anomalies (R; dysplasia, hydronephrosis, horseshoe kidney, and ectopic kidney), 50% to 80%, and limb defects (L; radial aplasia, polydactyly, syndactyly, and other limb anomalies), 40% to 50% [1,2,3]. The outcome of VACTERL depends on the severity of the associated abnormalities. It is thought that defective mesodermal development of unknown origin causes VACTERL. Genetic involvement is unknown; the majority of cases are sporadic [1,2,3]. Most infants with VACTERL require surgical and rehabilitative interventions. Moreover, VACTERL’s association with hydrocephalus (VACTERL-H), which is probably secondary to aqueductal stenosis, is much rarer and has a poorer prognosis than any other association [4,5,6]. Only a few patients have survived, most of whom have considerable neurodevelopmental and physical handicaps [4,5,6]. Previous reports have suspected that the combination of VACTERL anomalies with hydrocephalus might be associated with autosomal-recessive or X-linked inheritance. However, like other VACTERL associations, the etiology and heredity of the VACTERL-H association are still unclear.

Here, we report the prenatal ultrasonographic findings and postnatal three-dimensional computed tomographic (3D CT) findings of a VACTERL-H association case. To the best of our knowledge, this is the first report of a dizygotic twin with a nonfamilial VACTERL-H association diagnosed prenatally using detailed ultrasound examinations and fetal echocardiography and confirmed with postnatal 3D CT findings and neonatal echocardiography.

## 2. Case

A 34-year-old multiparous woman was referred to our hospital for suspected fetal ventriculomegaly at 31 + 3 weeks’ gestation. She had a healthy 6-year-old son without any congenital abnormalities. Her second pregnancy was conceived by in vitro fertilization and embryo transfer (IVF-ET). At 24 + 0 weeks gestation, routine ultrasound examinations at a local clinic revealed hydrocephalus in one of the twins. The other twin had no definite structural anomalies on ultrasound examinations. The pregnant woman was transferred to our clinic at 31 + 3 weeks gestation.

**Figure 1:**
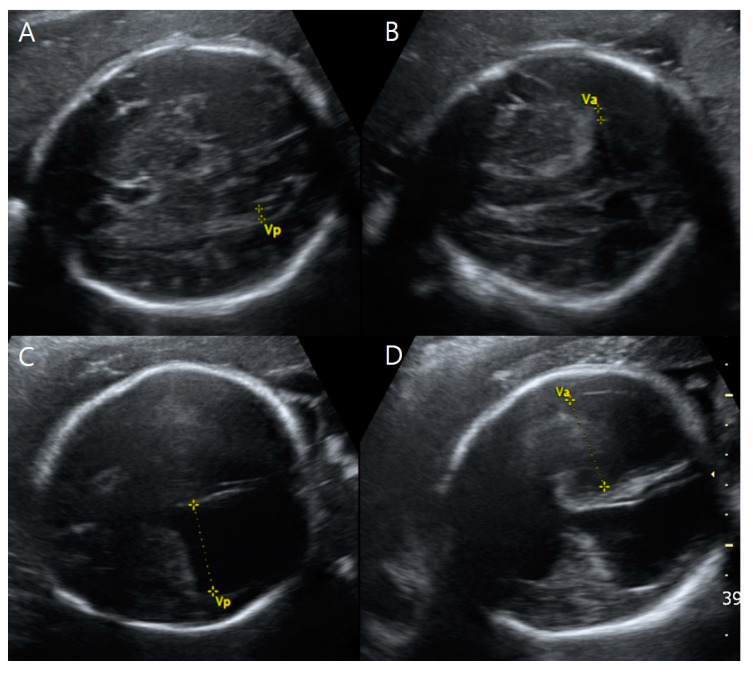
Transventricular view of the twin fetuses on ultrasound examinations at 31 + 3 weeks gestation. (**A**,**B**) Fetus B, a healthy female with normal-sized atrial widths of both left and right lateral ventricles (Va and Vp were <10 mm) (**C**,**D**) Fetus A, a male with severe ventriculomegaly; left and right (Va, Vp) at 31.16 mm and 29.59 mm, respectively. Note that in Va and Vp, the abbreviation ‘a’ indicates the closer side to the ultrasound probe (the anterior side of the mother), and the abbreviation ‘p’ presents the farther side from the ultrasound probe (the posterior side of the mother).

**Figure 2:**
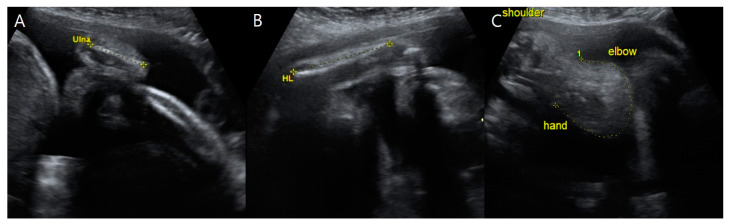
Upper-limb anomalies of fetus A on ultrasonography at 31 + 3 weeks gestation. (**A**) The length of fetus A’s ulna was 2.63 cm (<5th percentile). (**B**) Fetus A’s humeral length was 4.63 cm (<5th percentile). (**C**) Fetus A’s forearm with the absence of radii, thumbs, and radial clubhands, or abnormally curved hands toward the absent bone.

**Figure 3:**
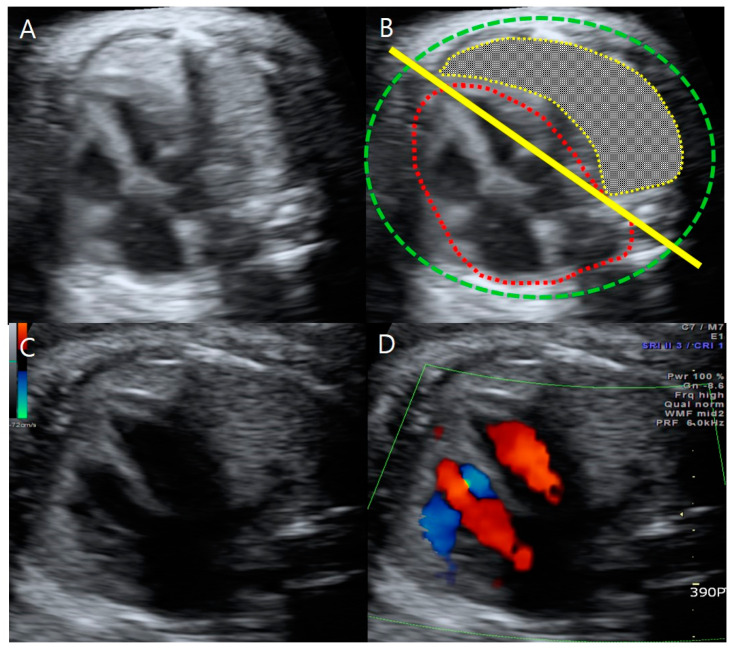
A four-chamber view of fetus A at 31 + 1 weeks gestation. (**A**) Right lung agenesis with dextrocardia. (**B**) Diagram of right lung agenesis and dextrocardia. Green dot: thorax, red dot: heart, yellow dot: left lung, yellow line: a connecting line from the spine (posterior part) to the sternum (anterior part). (**C**) Right atrial enlargement of fetus A. (**D**) Right atrial enlargement viewed with Doppler sonography in the absence of other major cardiac defects, such as valvular abnormalities.

**Figure 4:**
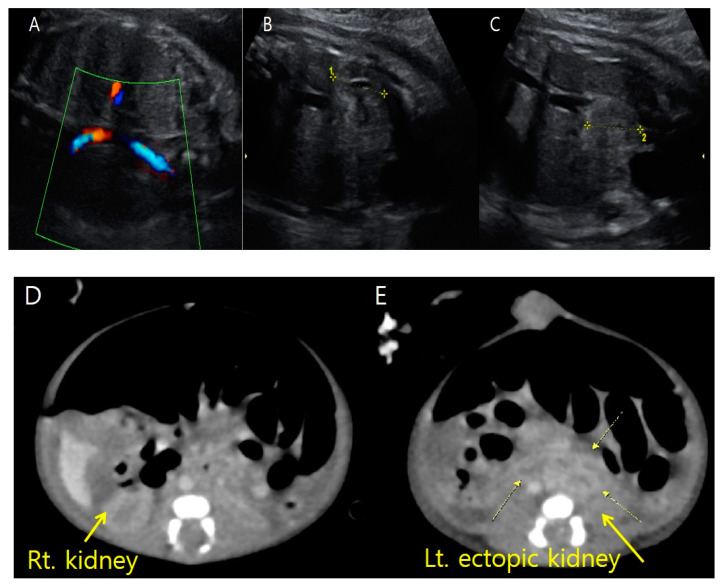
Prenatal ultrasonography at 31 + 1 weeks gestation and postnatal abdominal three-dimensional computed tomography show fetus A’s ectopic left kidney. (**A**) On color Doppler ultrasonography, there was no definite renal flow to the left usual renal fossa. (**B**) Fetus A’s right kidney is relatively small (2.26 cm in length) but located in the usual position. (**C**) Fetus A’s ectopic left kidney is also relatively small (2.27 cm in length) and located almost adjacent to the bladder. (**D**) The right kidney is in the regular position. (**E**) The ectopic left kidney lies adjacent to the bladder and anterior to the spine.

**Figure 5:**
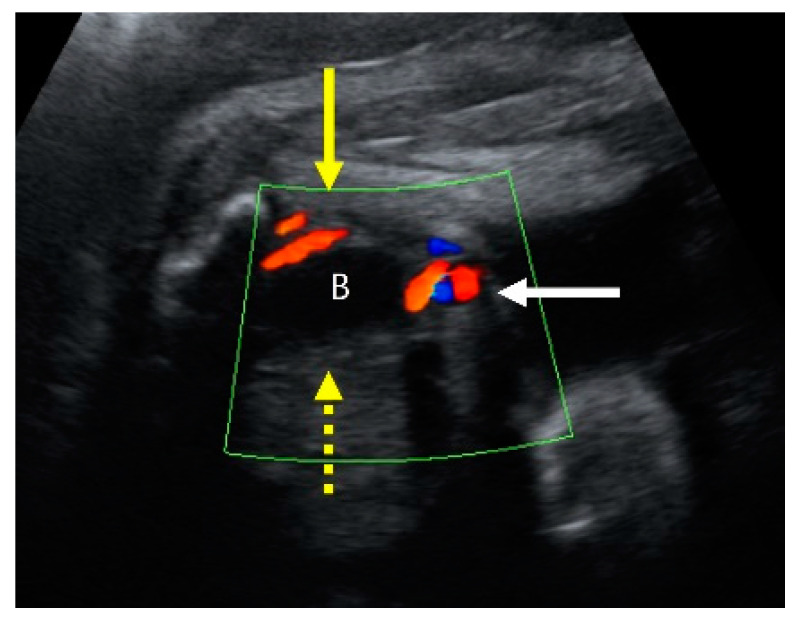
A transverse view of prenatal ultrasonography at 31 + 1 weeks’ gestation shows a single umbilical artery (SUA) of fetus A. Color Doppler shows the bladder section (B: bladder), with one side of umbilical artery (yellow arrow) and the other side missing (yellow dotted arrow). The white arrow indicates the umbilical cord, containing vessels.

**Figure 6:**
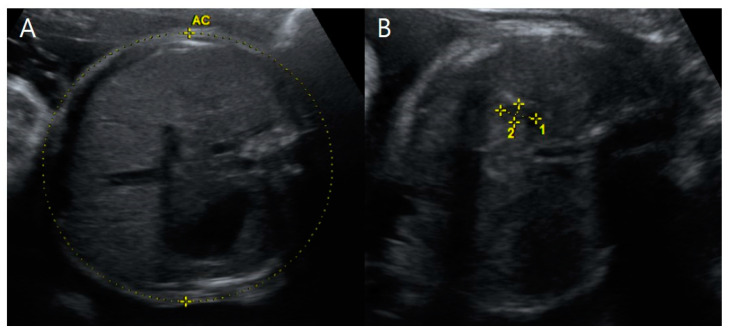
Transverse view of prenatal ultrasonography at 31 + 1 weeks gestation. (**A**) Fetus B, a healthy female with a normal stomach filled with amniotic fluid. (**B**) Fetus A, an affected male with a small stomach sized 0.91 × 0.47 cm^2^ accompanied by polyhydramnios and without time variance, highly suggesting tracheoesophageal fistula. Note that the abnormally increased amniotic fluid is not shown in this figure.

**Figure 7:**
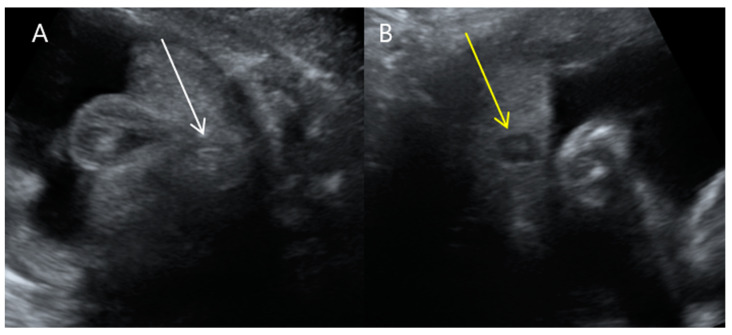
Transverse views of prenatal ultrasonography at 31 + 1 weeks gestation. (**A**) Anorectal atresia in fetus A with a sharp-ended oval shape slightly displaced to the right (white arrow). (**B**) Normal anus in fetus B with fried egg target sign (yellow arrow).

Targeted ultrasound examinations showed a dichorionic-diamniotic twin pregnancy with a definite discordance in estimated fetal weight (EFW) between the first (fetus A, male) and the second (fetus B, female) fetuses. The twins were highly suspected to be dizygotic because they are different sexes. The male fetus weighed 1203 g at 31 + 3 weeks gestation (10th percentile at 32 weeks’ gestation-1310 g), with the single-deepest pocket of amniotic fluid measuring 10 cm. The female twin weighed 1595 g at 31 + 3 weeks gestation, with the single-deepest pocket of amniotic fluid measuring 4 cm. The twins also showed discordance in structural abnormalities. Compared with fetus B, fetus A presented not only with hydrocephalus (Figure 1) but also bilateral dysplastic upper arms, radial aplasia (Figure 2), right pulmonary agenesis (Figure 3), interrupted inferior vena cava (IVC), a unilateral hypoplastic ectopic kidney (Figure 4), a single umbilical artery (Figure 5), a tracheoesophageal fistula with small stomach (Figure 6), polyhydramnios, and anal atresia (Figure 7).

On transventricular view, both of fetus A’s lateral ventricles were severely dilated at 31.0 mm and 29.5 mm, resulting in cortical mantle thinning (Figure 1). Fetus A’s third ventricle was dilated to 5.8 mm, but no other brain abnormalities were found.

A detailed sonographic examination of fetus A detected a bilateral absence of the radii and thumbs (Figure 2). Both hands deviated in the direction of the absent bone, leading to an abnormally curved posture and radial clubhands. For the right forearm, we could not find the humerus because there was only one long bone proximally adjacent to the shoulder and distal to the curved hand.

On a four-chamber view of fetus A, we noted the complete displacement of the heart to the right side and right atrial enlargement without major cardiac defects, such as septal defects, anomalies of the great vessels, or valvular abnormalities (Figure 3). This cardiomediastinal shift towards the absent right lung without a contralateral chest mass and with an intact diaphragm was consistent with the finding of right pulmonary agenesis, which was confirmed postnatally. Although an azygos continuation to the superior vena cava could not be found, an interrupted inferior vena cava was suspected because it was hard to detect the IVC in its usual position, right anterior to the aorta, in fetus A on a transverse view of the fetal upper abdomen, which should include the stomach on the left, the liver on the right, the aorta, and the inferior vena cava (IVC) located anterior to the spine in a normal fetus.

Fetus A’s right kidney was located in the normal renal fossa. Although fetus A’s right kidney was relatively small, it showed normal corticomedullary differentiation without structural abnormalities. On coronal view exams using color Doppler ultrasonography, we found only the right renal artery originating from the descending aorta. To rule out unilateral renal agenesis, we scrutinized the entire pelvic area and finally found the left kidney. However, the left kidney was also relatively small and displaced to an ectopic site almost adjacent to the bladder and near the midline of the body (Figure 4). A single umbilical artery was observed in fetus A (Figure 5).

**Figure 8:**
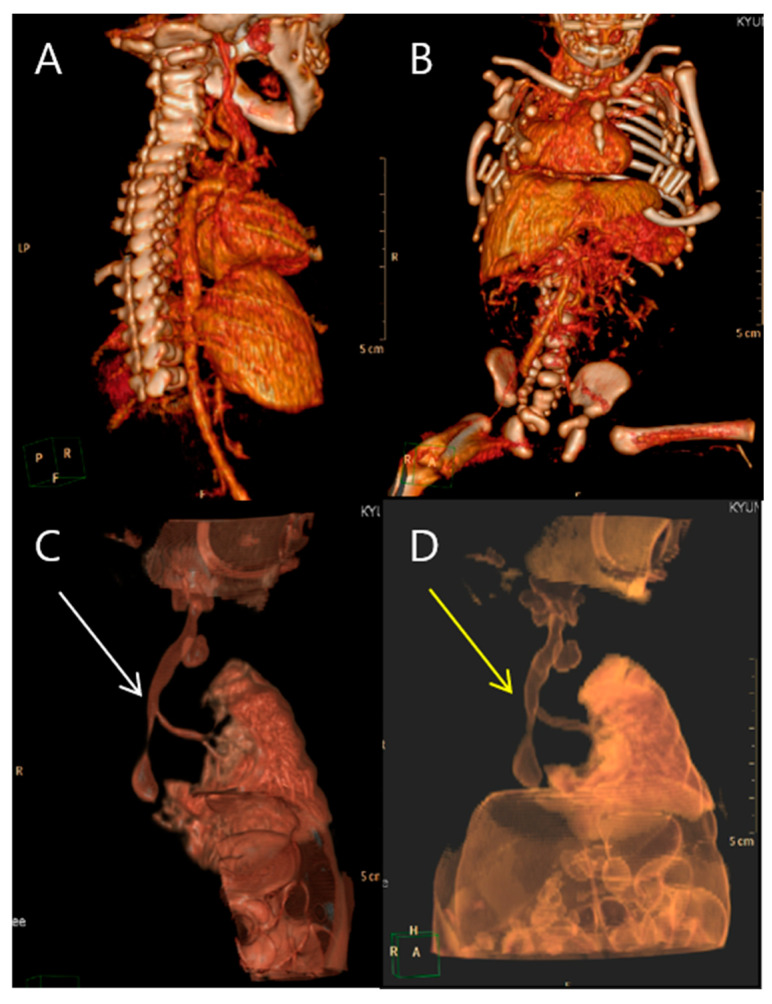
3D cardiac angio computed tomography of fetus A. (**A**) Dextrocardia of embryonic arrest with pulmonary hypoplasia from posterior to anterior coronal view. (**B**) Dextrocardia of embryonic arrest with pulmonary hypoplasia from anterior to posterior coronal view. (**C**) Tracheoesophageal fistula with right bronchus (white arrow). (**D**) Tracheoesophageal fistula with right bronchus (yellow arrow).

On the transverse view, we noted a small stomach in association with polyhydramnios in fetus A. These were crucial findings from the prenatal ultrasound examinations for the diagnosis of tracheoesophageal fistula (TEF), which were confirmed by 3D CT (Figure 6 and Figure 8). Compared with the normal-sized stomach filled with amniotic fluid in fetus B, fetus A’s stomach size did not increase over time, as shown by repeated exams (Figure 6).

In fetus A, we did not observe the hyperechoic mucosa within the hypoechoic ring that we found in fetus B (Figure 7). Instead, a sharp-ended, oval-shaped, relatively long, and thin-shaped anus was noted at the site, slightly deviated from midline to the right side, suggesting that anorectal atresia was complicated in fetus A, which was also confirmed postnatally (Figure 7).

Based on the malformations that we found, including hydrocephalus, bilateral dysplastic upper arms, radial aplasia, unilateral pulmonary agenesis, interrupted inferior vena cava, a unilateral hypoplastic ectopic kidney, a single umbilical artery, a tracheoesophageal fistula with a small stomach, polyhydramnios, and anal atresia, we diagnosed VACTERL-H in a dizygotic twin in utero.

The mother of the dizygotic twin pregnancy had preterm labor (PTL) accompanied by dyspnea. She was admitted to the hospital at 31 + 5 weeks gestation for amnioreduction, karyotyping to rule out chromosomal abnormalities, and infection studies to exclude infectious diseases. Amnioreduction relieved the mother’s dyspnea, which might have been caused by polyhydramnios on the affected twin’s side. After the procedure and short-term tocolytics to manage the PTL, the pregnant woman was discharged. The results of the index patient’s karyotyping were normal (46, XY), as were the findings from the infection studies of the maternal serum and amniotic fluid. The fetus B without structural anomalies showed a normal karyotype (46, XX). Based on karyotyping results, the twins were considered dizygotic.

**Figure 9:**
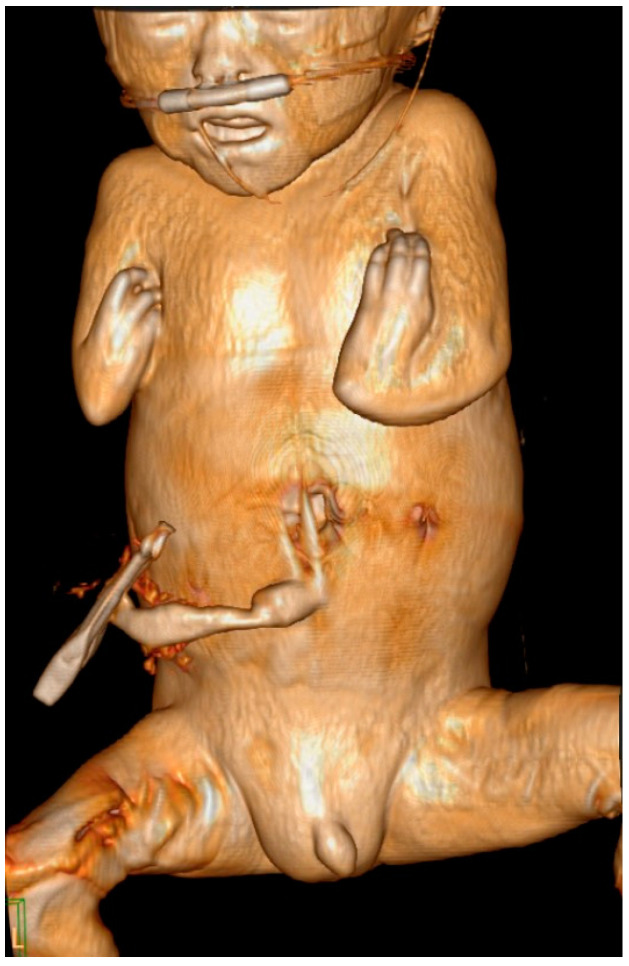
Upper-limb deformity with bilateral dysplastic upper arm and hypoplastic thumb on postnatal 3D computed tomography.

**Figure 10:**
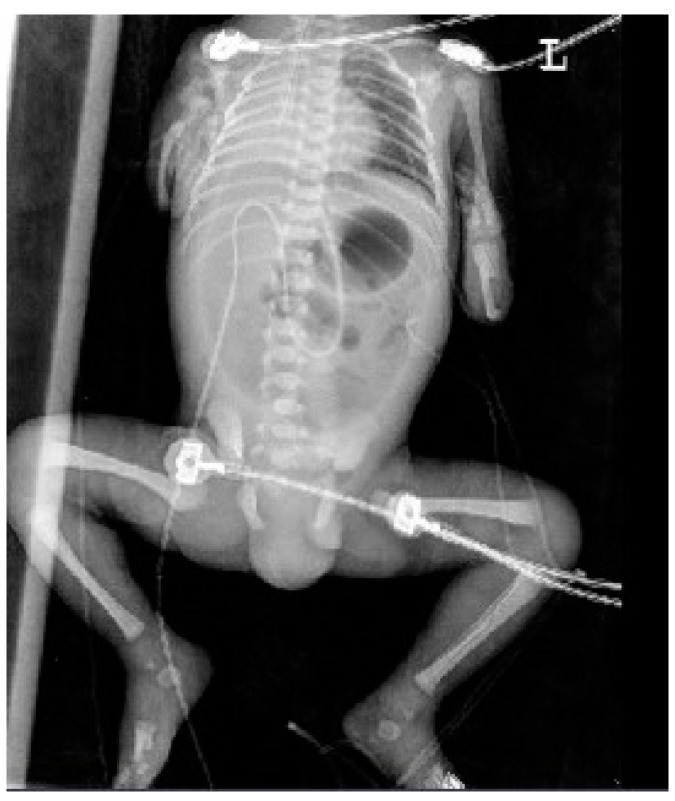
Infantogram of fetus A showing upper-limb dysplasia, pneumoperitoneum, and total atelectasis of the right lung.

**Figure 11:**
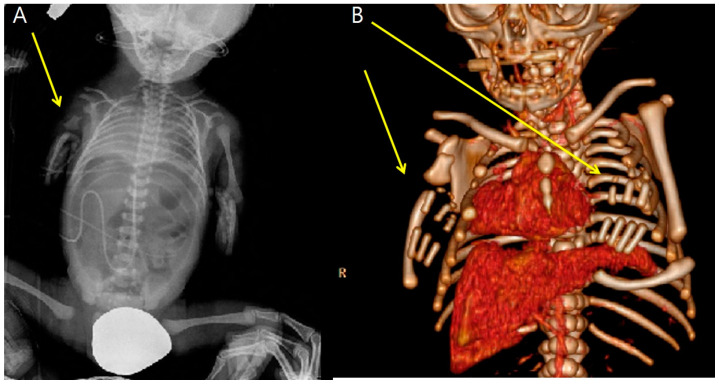
Infantogram and 3D computed tomography showing upper-limb deformity. (**A**) Infantogram representing aplasia of the right proximal humerus and bilateral hypoplastic upper arm (yellow arrow). (**B**) 3D computed tomography representing bilateral hypoplastic thumbs (yellow arrows).

**Figure 12:**
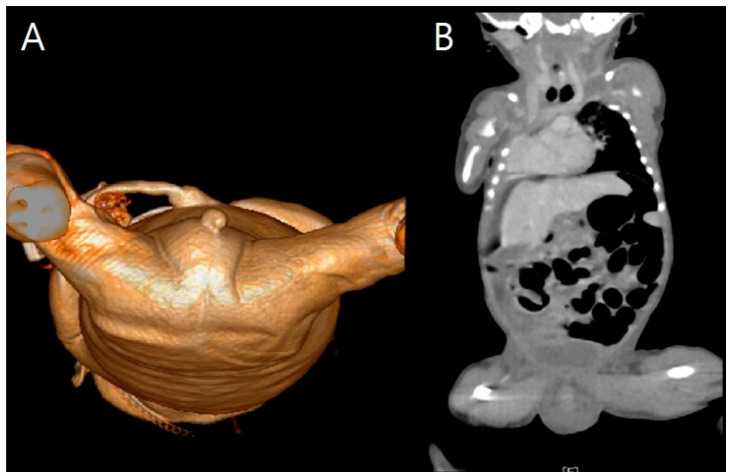
Abdominal pelvic computed tomography revealed imperforate anus and colon dilatation in fetus A. (**A**) Imperforate anus from caudal to cranial view. The position of the anal dimple was slightly displaced to the right. (**B**) Dilated colon from coronal view.

At 34 + 0 weeks gestation, an emergency cesarean delivery was performed because of relapsed preterm labor. The first baby was a male with VACTERL-H association, weighing 1390 g, and the second baby was a healthy female, weighing 1890 g. The Apgar scores at 1 and 5 min were 6 and 8 in the first twin baby and 5 and 8 in the second twin baby. The first twin had multiple anomalies diagnosed prenatally. Related imaging studies were performed, including an infantogram and 3D CT (Figure 8, Figure 9, Figure 10, Figure 11 and Figure 12). A cardiacangio 3D CT revealed dextrocardia of embryonic arrest with pulmonary hypoplasia and a tracheoesophageal fistula with a right bronchus (Figure 8). The infantogram and 3D CT showed upper-limb agenesis, which aligned with the prenatal diagnosis, including aplasia of the right proximal humerus, radial hypoplasia, and hypoplastic thumb (Figure 9, Figure 10 and Figure 11). The first twin baby also had an imperforate anus and colon dilatation (Figure 12). Neonatal echocardiography showed dextrocardia with right atrial enlargement and an interrupted inferior vena cava without major congenital cardiac abnormalities. The findings from postnatal imaging supported the prenatal diagnosis of a VACTERL-H association. On his fifth day of life, the twin baby with the VACTERL-H association died.

## 3. Discussion

**Figure 13:**
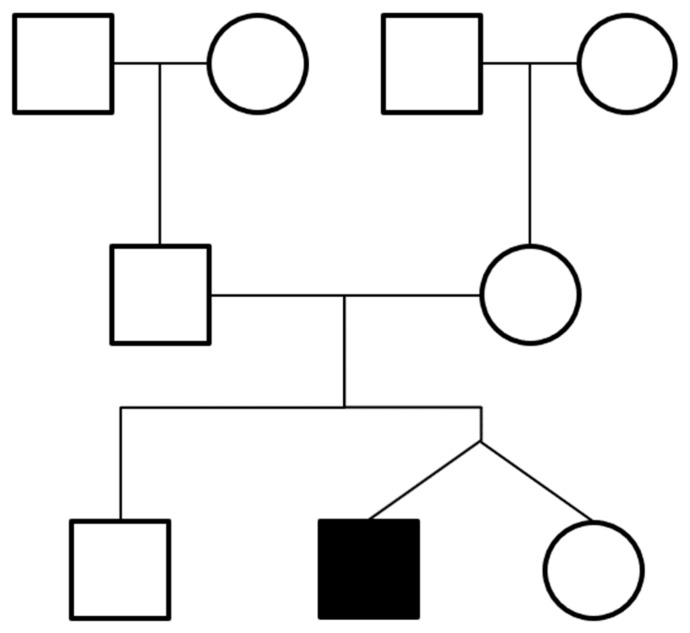
Pedigree analysis. The affected twin baby had a normal older brother and normal twin sister, as well as parents and grandparents without VACTERL-H association-related congenital anomalies.

The principal finding of this report is that a dizygotic twin was prenatally diagnosed and postnatally confirmed as having a VACTERL-H association. The major prenatally and postnatally confirmed findings were anal atresia, interrupted inferior vena cava, unilateral pulmonary agenesis, tracheoesophageal fistula, unilateral hypoplastic ectopic kidney, a single umbilical artery, bilateral dysplastic upper arms, radial aplasia, and hydrocephalus. The other twin and the twin’s 6-year-old brother were healthy. Additionally, we did not find any congenital abnormalities related to the VACTERL-H association in a pedigree analysis based on interviews (Figure 13). In previous case reports, the VACTERL-H association was related to X-linked or autosomal recessive inheritance [4,5,6]. However, there is a paucity of information about genetics in the VACTERL-H association, and most cases are reported as sporadic [7,8,9,10,11].

In this case, we did not identify vertebral anomalies, which are the most frequently found features of VACTERL-H and include transitional vertebrae, hemivertebrae, block vertebrae, scoliosis, spina bifida, butterfly vertebrae, and vertebral agenesis. However, vertebral anomalies are not an indispensable component of VACTERL-H and are reported in approximately 60–95 percent of affected patients [12]. Although renal, limb, and vertebral anomalies are most commonly identified, VACTERL-H association remains a diagnosis of exclusion and should be considered in cases of at least three core abnormalities: vertebral defects (V), anal atresia (A), cardiac defects (C), tracheoesophageal fistula (TE), renal anomalies (R), and limb defects (L) [1,2,3]. We acknowledge the potential hypothesis that imaging techniques such as infantograms and 3D CT might fail to diagnose subtle vertebral anomalies and that autopsies should be analyzed in future studies.

As in our case, progressive ventriculomegaly serves as a milestone for the prenatal diagnosis of the VACTERL-H association. However, ventriculomegaly is not a prominent feature before 19 weeks gestation, so the diagnosis of VACTERL-H association in an early trimester could be hard. [13] In our case, ventriculomegaly was identified at a local clinic on routine prenatal examination at 24 + 0 weeks gestation. After the pregnant patient was transferred to our hospital, we detected severe bilateral ventriculomegaly at 31 + 3 weeks gestation. In addition to detailed ultrasound examinations of the brain, we tried to perform fetal magnetic resonance imaging (MRI) to evaluate for brain abnormalities with more precision, but the woman pregnant with twins and experiencing polyhydramnios could not lie on her back. In future studies, fetal MRI should be considered to assess for other brain anomalies when severe ventriculomegaly-lateral ventricular dilatation greater than 15 mm is encountered.

We found fetus A to have a small stomach with polyhydramnios in prenatal ultrasonography. Polyhydramnios is the most common ultrasonographic clue to TEF, though polyhydramnios is non-specific and present in about 60 percent of pregnancies [14]. Once polyhydramnios and/or fetal airway obstruction are suspected, clinicians should focus on stomach size in the transverse view of prenatal ultrasound examinations and the variance in stomach size over time, considering gastric emptying time because a small stomach bubble with normal amniotic fluid volume is often a transient finding in a normal fetus. Although stomach size assessment can be subjective, an anomalous fetus will occasionally be missed if very small stomach bubbles are considered normal. Postnatal CT or MRI can confirm the diagnosis and site of obstruction, and, in our case, postnatal 3D CT findings allowed for the diagnosis of TEF in VACTERL-H.

According to the literature, a detailed examination of the upper limbs in the first-trimester screening may be useful for detecting familial VACTERL-H cases. Approximately 40–50 percent of VACTERL-associated patients had limb malformations such as thumb aplasia or hypoplasia, polydactyly, and lower-limb anomalies [15,16]. We detected upper-limb anomalies, including radial aplasia, syndactyly, and clubhands, on prenatal ultrasound examinations. It is important to evaluate the length, shape, and movement of long bones, including the radius, ulna, tibia, and fibula, as well as the femur and humerus.

In prenatal sonography, a normal anus looks like a fried egg surrounded by the anal sphincter called the target sign. In this case, the anal atresia appeared as a sharp-ended oval shape without any visible anal mucosa located close to the genitalia and slightly laterally deviated [17]. Anorectal malformations (ARMs) are relatively common congenital anomalies, but the coexistence of the VACTERL association significantly worsens the clinical and surgical outcomes of patients with ARMs. With the VACTERL-H association, a colostomy was the most frequent surgical treatment, but its clinical outcome was poor [18].

Cardiac abnormality is a characteristic finding in VACTERL-H, as mentioned. In terms of embryology, cardiac and pulmonary genesis begins as early as the third week of gestation. The association of pulmonary hypoplasia with VACTERL-H is very rare in clinical practice and increases morbidity [19]. Our patient showed no major cardiac defects and had right atrial enlargement but an interrupted IVC. There have been a few cases of unilateral pulmonary agenesis detected prenatally [19]. Importantly, right pulmonary agenesis was detected in our case based on a cardiomediastinal shift towards the absent lung without a contralateral chest mass or diaphragmatic hernia on prenatal ultrasound examinations. Pulmonary agenesis could contribute to a higher risk of respiratory failure in the event of a respiratory infection because patients with VACTERL-H have a reduced pulmonary reserve [20,21]. When unilateral pulmonary agenesis is suspected, we recommend color Doppler to confirm the absence of the ipsilateral pulmonary artery and to find associated vascular anomalies.

According to the previous literature, the VACTERL-H association has a very poor prognosis prenatally and/or postnatally. Some fetuses were terminated selectively, and others were stillborn or died during the neonatal period. However, it was possible to observe the natural progression of a fetus with VACTERL-H association without termination of pregnancy in our case because of the dizygotic twin pregnancy with one affected twin and one normally developing twin, the discordance both in size and congenital anomalies, and, moreover, the accurate diagnosis is made after 24 weeks of gestation. Importantly, the International Society of Ultrasound in Obstetrics and Gynecology (ISUOG) recommends that the first and second trimester screenings be performed in twin pregnancies [22]. The prenatal diagnosis of the VACTERL-H association early in pregnancy might be challenging. However, considering the adverse outcomes of the VACTERL-H association and neonatal mortality, prenatal diagnosis should be a focus of future studies. The range of detailed findings, in this case, might contribute to an earlier and more precise prenatal diagnosis.

## 4. Conclusions

This report is the first of the VACTERL-H association diagnosed using prenatal ultrasonography and postnatal 3D CT in a dizygotic twin. When fetal structural abnormalities, such as ventriculomegaly, a small stomach with polyhydramnios, an abnormally positioned heart, and increased amniotic fluid, are observed on routine prenatal ultrasound examinations, we suggest a systemic evaluation from head to toe of the affected fetus to find out about other complex abnormalities, leading to the diagnosis of VACTERL-H association, a multisystem congenital malformation. The findings of the present study might contribute to a diagnosis in an earlier period of pregnancy.

## Data Availability

The data presented in this study are available on request from the corresponding author. The data is not publicly available due to privacy and ethical concerns.
